# Anastomotic occlusion after laparoscopic low anterior rectal resection: a rare case study and literature review

**DOI:** 10.1186/s12957-022-02610-5

**Published:** 2022-05-06

**Authors:** Chunhai Hu, Hui Zhang, Lingpeng Yang, Jian Zhao, Qiang Cai, Long Jiang, Lin Meng, Zhi Wang, Zhengrong Wen, Yunhua Wang, Zhiyong Yu

**Affiliations:** 1 Department II of Hepatobiliary Surgery, The People’s Hospital of Chuxiong Yi Autonomous Prefecture, The Fourth Affiliated Hospital of Dali University, Chuxiong, China; 2 Department of Hepatobiliary and Pancreatic Surgery, The Affiliated Hospital of Yunnan University, The Second People’s Hospital of Yunnan Province, Kunming, Yunnan Province China

**Keywords:** Low anterior rectal resection, Anastomotic occlusion, Therapeutic regime

## Abstract

**Background:**

With the development of laparoscopic techniques and the broad clinical application of various anastomotic types, anal-preserving low anterior rectal resection and ultra-low anterior rectal resection have been popularized. Some patients with rectal cancer have retained their anus and improved their quality of life. Nevertheless, the incidence of postoperative anastomotic stenosis remains high, and anastomotic occlusion is even rarer.

**Case presentation:**

We report a case of anastomotic occlusion in a patient with rectal cancer, which occurred after undergoing laparoscopic low anterior rectal resection + prophylactic terminal ileal fistulation at our department. Under endoscopy, we used a small guidewire to break through the occluded anastomosis, thereby finding the lacuna. After endoscopic balloon dilation, digital anal dilatation, and continuous dilator-assisted dilation, the desired efficacy was achieved, ultimately recovering ileal stoma. Postoperative follow-up condition was generally acceptable, without symptoms like abdominal pain, bloating, or difficulty in defecation.

**Conclusion:**

Numerous factors cause postoperative anastomotic stenosis in patients with rectal cancer. Complete occlusion of anastomosis occurs relatively rare in clinical practice, and is challenging to treat. This case was our first attempt to remove the anastomotic occlusion successfully, which avoided re-operation or pain from the permanent fistula.

## Background

With the application and popularization of laparoscopic techniques and anastomosis, laparoscopic surgery has become the preferred treatment for rectal cancer. It has the advantages of a clear surgical field, better convenience than open surgery, low invasiveness and fast recovery. Anastomotic fistula and stenosis are major complications following colorectal surgery [1, 2]. The incidence of anastomotic stenosis after colorectal cancer surgery is 3–30% [3]. Despite the rarity, complete occlusion of anastomosis has been reported in some populations [4]. Herein, we report a case of anastomotic occlusion after laparoscopic low anterior rectal resection + prophylactic terminal ileal fistulation, which was diagnosed and treated at our department. After rectal cancer surgery, the causes and treatments of anastomotic stenosis are reviewed with the previous literature reports.

## Case presentation

A 53-year-old female patient was hospitalized at our department on August 21, 2020 because of the “rectal mass found 7 days ago.” Digital anal examination findings (chest-knee position): a hard mass of approximately 3 × 3 cm in size was palpable at the 7–9 o’clock orientation 4 cm apart from the anus, with no tenderness and moderate mobility. No blood stain was detected when exiting the finger cot, and the tumor markers were normal. Abdominal CT and pelvic MRI findings: space-occupying lesions in the lower rectum. Preoperative diagnosis: “moderately differentiated adenocarcinoma of the lower rectum(T3N0M0)”. On August 31, 2020, the patient received “laparoscopic low anterior rectal resection + prophylactic terminal ileal fistulation" under general anesthesia. After the surgery, the anastomosis was unobstructed based on digital detection, about 2.8 cm in diameter and 3 cm away from the anus, with circular anastomotic nails palpable. Postoperative pathological diagnosis: moderately differentiated adenocarcinoma of the lower rectum. Good postoperative recovery was achieved without developing complications such as anastomotic leakage, stenosis, or bleeding. The patient refused chemotherapy due to personal reasons. She was told to receive recovery of small intestinal stoma 3 months later, during which regular follow-up visits were required.

**Fig. 1:**
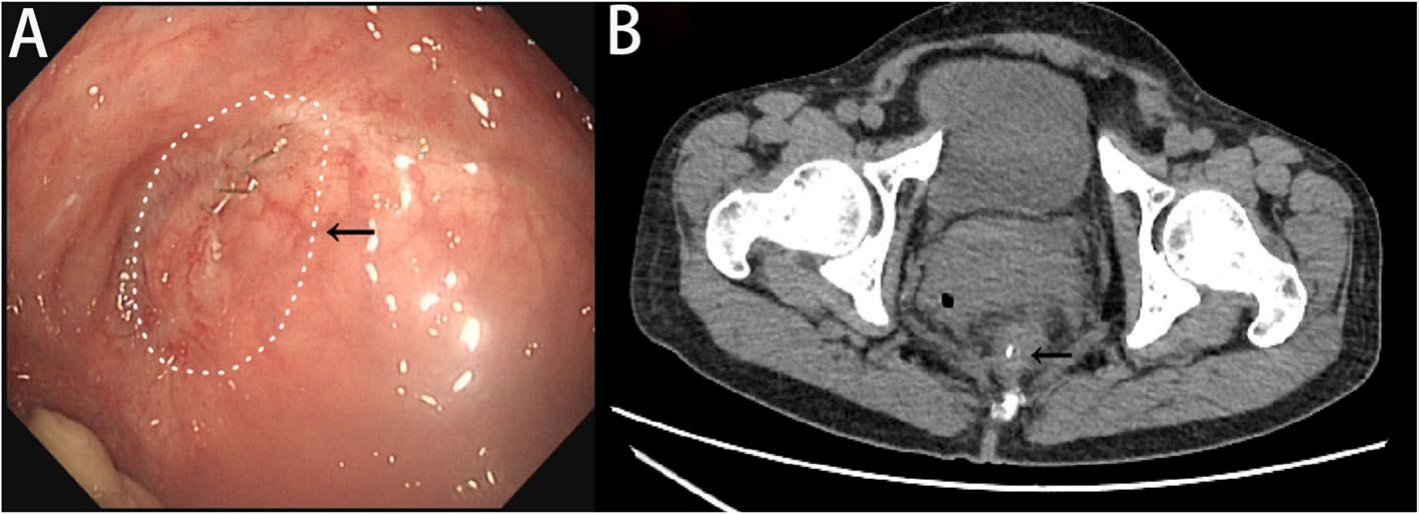
Colonoscopy and abdominal CT examination revealed that the rectal anastomosis was already occluded, and some anastomotic metal nails were observed at the stoma site

**Fig. 2:**
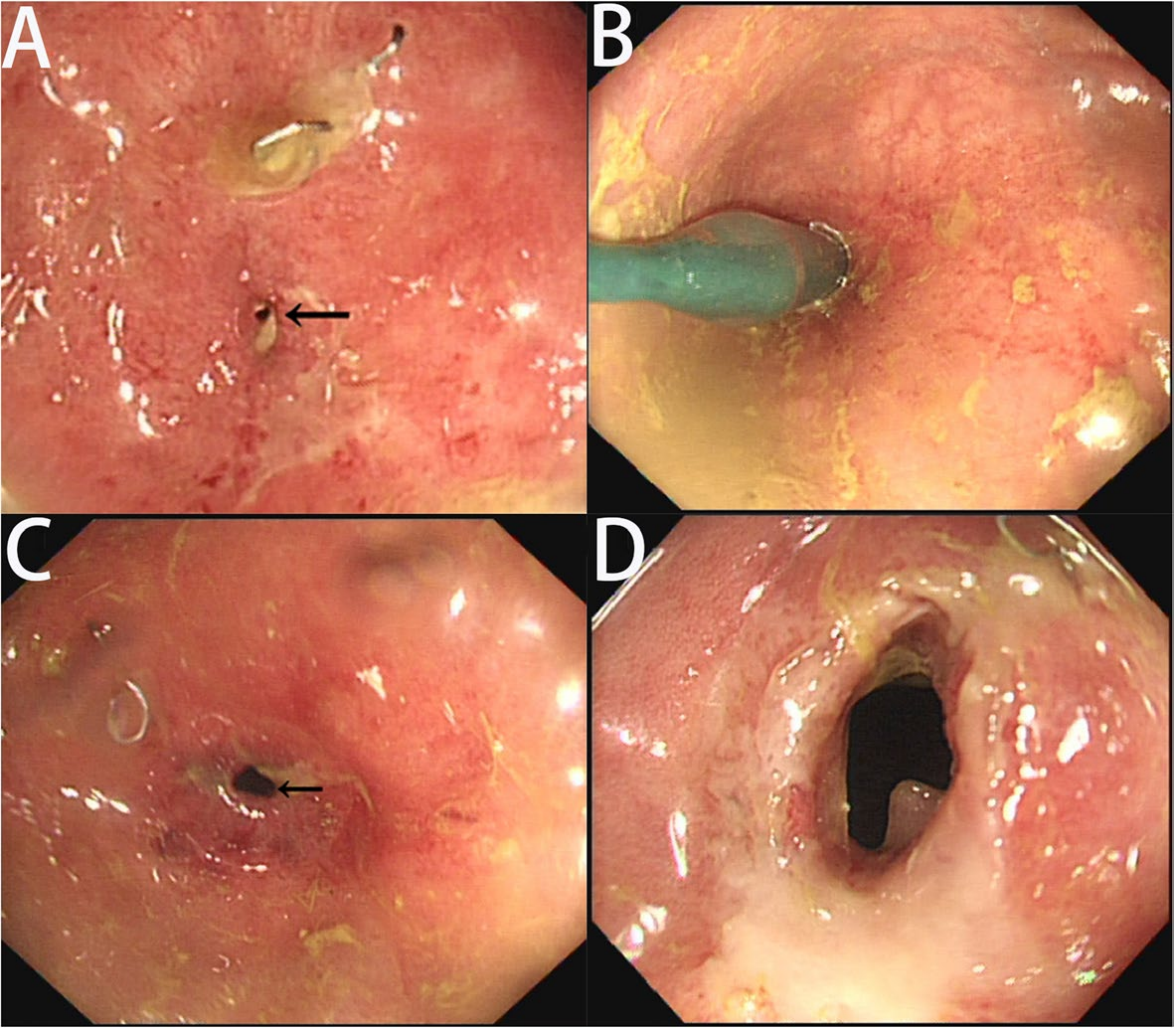
**A** A small anastomotic space was visible after puncturing the occluded anastomosis with a thin guide wire under endoscopy. **B** Using a guidewire, anastomosis was dilated with a balloon under endoscopy. **C** After initial dilation, the diameter of the anastomosis was approximately 0.3 cm. **D** After multiple times of endoscopic balloon dilation, the anastomosis was dilated to 0.8 cm in size

**Fig. 3:**
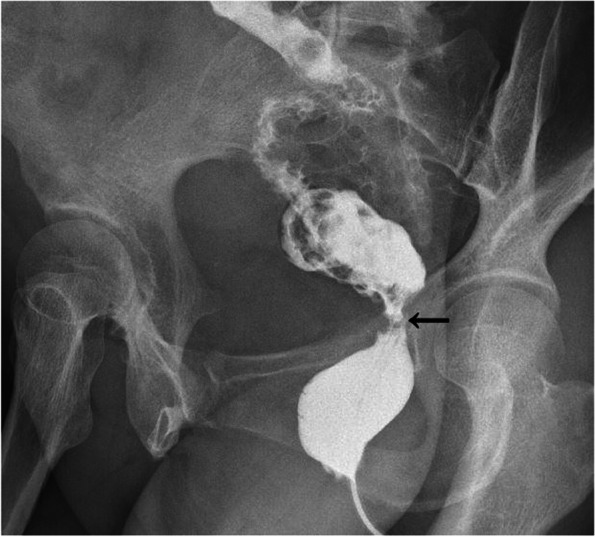
Rectal iodine contrast examination revealed stenosis of the rectal anastomosis

**Fig. 4:**
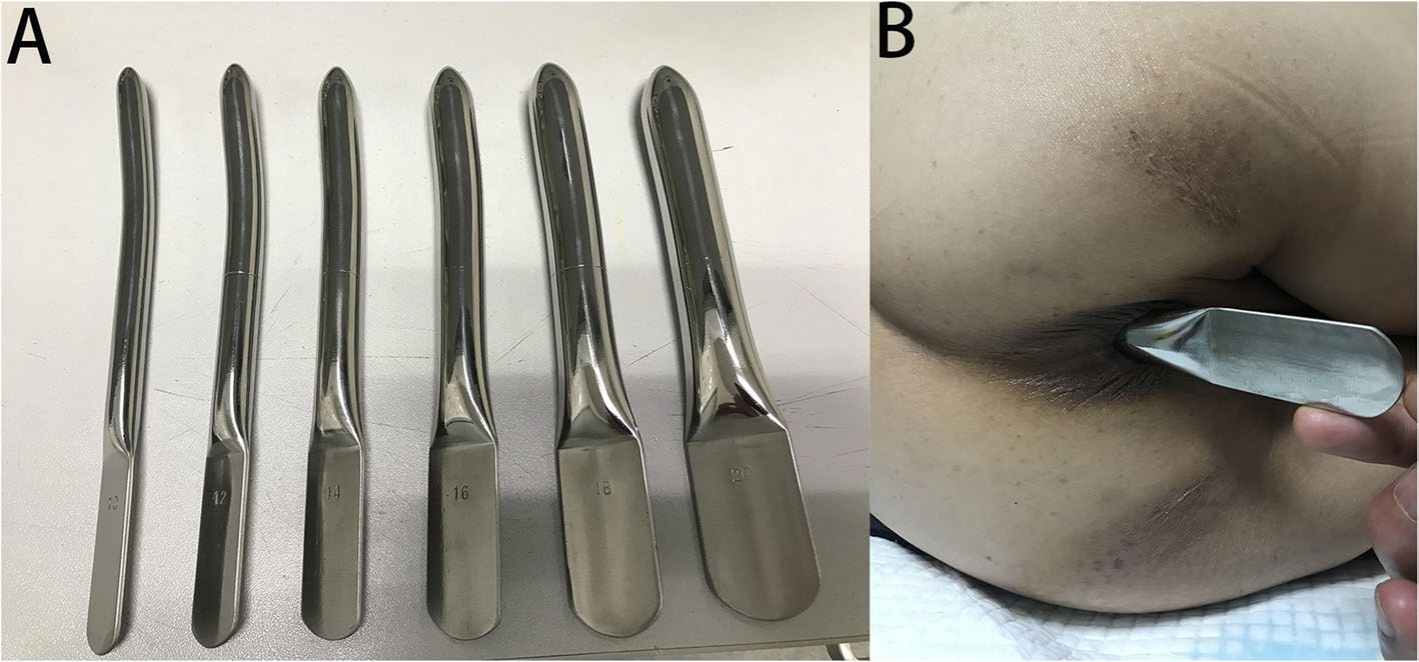
A Different types of metal anal dilators. B The rectal anastomosis was dilated for the patient using a 25-mm-diameter metal anal dilator

**Fig. 5:**
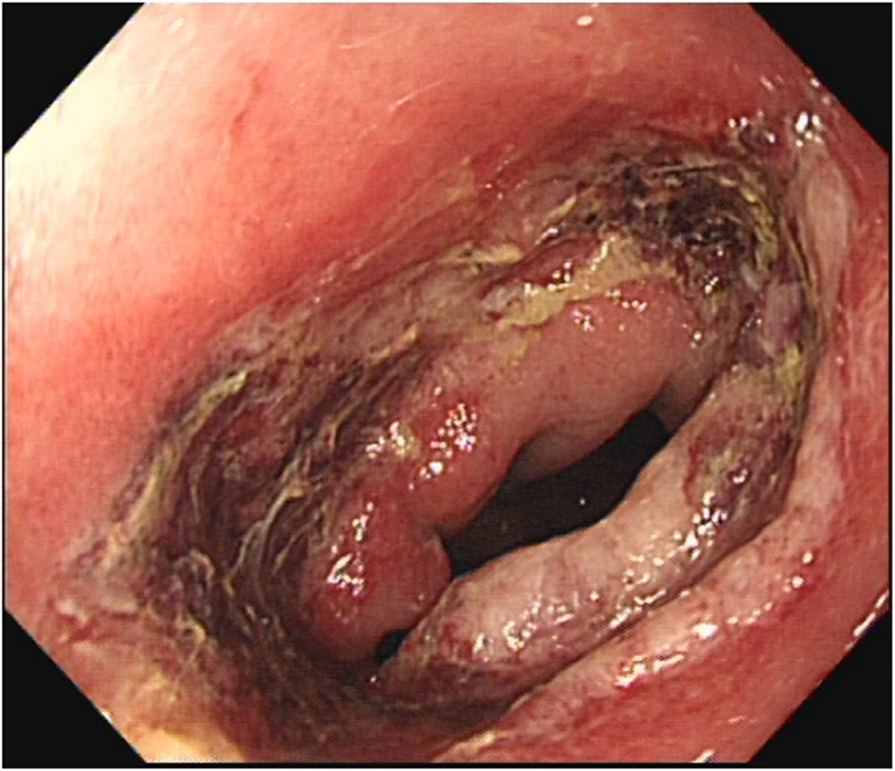
Colonoscopy showed that the rectal anastomosis was unobstructed, approximately 25 mm in diameter

After hospital discharge, the patient did not return for re-examination. On December 1, 2020, she visited our department and requested a small intestinal stoma recovery. Digital anal examination revealed a blind cavity at the rectal end, with undetectable anastomosis and palpable anastomotic nails. Colonoscopy and abdominal CT showed occluded anastomosis (Fig. 1A, B). We attempted to puncture the occluded anastomosis with a small guide wire under the endoscope. A small gap was observed after passing the guidewire (Fig. 2A). Furthermore, balloon dilation was performed with a guidewire (Fig. 2B), and the anastomosis was dilated to 0.3 cm in size (Fig. 2C). Later, endoscopic balloon dilation was performed multiple times, and the anastomosis was dilated to 0.8 cm (Fig. 2D). Whenever balloon dilation was performed, the patient would experience severe and unbearable pain in the anastomosis, and pain symptoms were not relieved after pain killer injection. Thus, the endoscopic dilation was terminated, and rectal imaging was performed (Fig. 3). Later, the right index fingertip was used to dilate the anastomosis, and the patient could tolerate pain. The dilation was performed 2–3 times per day for 2 consecutive weeks. The stoma was dilated to about 1 cm, which was far from satisfying the requirements of stoma recovery and defecation. Next, we used different models of anal dilators (diameters 10–25 mm) (Fig. 4A, B) for anal dilation. The patient presented with pain symptoms, which though could be tolerated. Initially, a 10-mm-diameter dilator was used to dilate the anus for 2–3 h once every 2–3 days, which lasted for 2 weeks. Next, the dilator was gradually replaced with larger diameter ones. The size of the anal dilator could be gradually enlarged according to the diameter of anastomosis. Three months later, a 25-mm-diameter dilator could easily enter the anastomosis, yielding satisfactory results. Upon enteroscopy reexamination, the anastomotic diameter was 25 mm (Fig. 5). The recovery of a small intestinal stoma was completed in this patient on April 2, 2021. We have followed this patient for 1 year after the surgery. During this period, the patient’s general condition was fine, without symptoms like abdominal pain, bloating, or difficulty in defecation, and she did not require a stool softener or any laxative medication. We will continue to follow up the patient for a long-time period.

## Discussion and conclusions

Anastomotic stenosis (AS) is one of the late complications following rectal cancer surgery. Very few patients develop anastomotic occlusion. Symptoms of AS usually include difficulty in defecation, bloating, and anal pain [5]. Causes of AS: (1) Surgeon’s experience: factors like the excessively small size of anastomotic, rectal division by multiple staplers, thick anastomotic intestine and high anastomotic tension can cause contraction of internal and external rectal sphincters, resulting in excessive proliferation of anastomotic fibrous tissues to form stenosis. (2) Anastomotic leakage and infection: they directly lead to the anastomotic scar healing and result in AS. (3) Anastomotic tissue ischemia: tissue ischemia is considered a possible risk factor for AS [6]. (4) Distance between lower tumor edge and anal edge: at low tumor positions, there is excessive tension and spasm of the anal sphincter, as well as edema and thickening of the intestinal wall, leading to AS. (5) Protective stoma: the anastomosis is in a disused state, lacking physical stimulation from feces, leading to AS. (6) Neoadjuvant chemo-radiotherapy: it has always been identified as a risk factor for anastomotic leakage and maybe eventually leads to AS. Using indocyanine green may reduce the rate of anastomotic leakage in colorectal surgery, even in particularly risky anastomoses [7]. (7) Recurrence of an anastomotic tumor. (8) Patient factors: general condition, gender, age, smoking, etc. For patients who smoke frequently, their blood vessels tend to contract due to arteriosclerosis, causing reduced tissue blood flow and cell hypoxia. The anastomotic healing fails because of ischemia and hypoxia, leading to anastomotic fistula or AS [8]. (9) Other factors: poor intestinal preparation, electric thermal injury, and the use of immunosuppressors. Anastomotic occlusion occurred in this patient is extremely rare. Reviewing the entire treatment process, we concluded that anastomotic ischemia caused by the low position of the rectal tumor and rectal division by 4 staplers was the primary cause of anastomotic occlusion. Another cause was the disused state of the anastomosis.

There are many treatments strategies for AS, including mechanical dilation, endoscopic therapy and transabdominal surgery [9–11]. These treatments are a gradual process rather than isolated from each other. (1) Mechanical dilation is the foremost method, the first-choice treatment for lower-position AS. Sloane et al. [9] reported that low rectal AS could be relieved or even removed by mechanical dilation. Finger anal dilatation is the simplest and most common method, although long-term persistence is required for alleviation. (2) Endoscopic therapy has been gradually applied to clinics in recent years, mainly balloon dilatation, self-expanding metal stenting and anastomotic radial incision. The advantages of balloon dilatation are minimal invasiveness and repeated applicability. It is suitable for patients with unreachable stenosis by digital rectal examination or with poor efficacy by finger dilation. If a single dilation is not satisfactorily effective, repeated colonoscopy operations are required, which do not have the effect of continuous dilation or relief [12]. For patients with failed balloon dilation, stenting can be considered. Stents can continuously support the stenosis section compared to balloon dilation, yielding a higher success rate. Nevertheless, it also has the risks and defects of high cost, stent displacement, and partial perforation [13, 14]. There have been reports on treating colorectal anastomotic stenosis with biodegradable stents. Further observation is required to confirm its long-term efficacy despite good preliminary efficacy [15]. For refractory AS with failed multiple balloon dilations or stent implantation, endoscopic electrocautery dilation is found effective, despite stenosis recurrence risk. (3) For patients with severe AS or non-surgical failure, surgical treatment is recommended, such as proximal neostomy, resection plus anastomosis and transanal radial incision. However, given their reoperation nature, there are probable risks of postoperative anastomotic leakage, bleeding, and restenosis, so careful choice is required.

The occurrence of AS is associated with numerous factors, where prevention should be emphasized. Once discovered, early treatment should be given. Complete occlusion is a rare condition that is difficult to manage. Improper management may exacerbate the patients' condition and economic burden. From simple to difficult and from non-invasive to invasive, the therapeutic strategy should depend on individual conditions [16–19]. Where necessary, multiple treatments can be used in combination. When conditions permit, novel therapeutic protocols can also be attempted upon patients’ consent. Our ultimate goal is to remove the occlusion and attain good efficacy.

## Data Availability

All data during the study are included within the article.

## Abbreviation

AS: Anastomotic stenosis
